# Enhancing linkage to HIV care in the “Universal Test and Treat” era: Barriers and enablers to HIV care among adults in a high HIV burdened district in KwaZulu-Natal, South Africa

**DOI:** 10.1186/s12889-023-16576-w

**Published:** 2023-09-09

**Authors:** Edward Nicol, Ngcwalisa Amanda Jama, Vuyelwa Mehlomakulu, Mbuzeleni Hlongwa, Desiree Pass, Wisdom Basera, Debbie Bradshaw

**Affiliations:** 1 Burden of Disease Research Unit, South African Medical Research Council, P.O. Box 19070, TygerbergCape Town, 7505 South Africa; 2 Division of Health Systems and Public Health, Stellenbosch University, Cape Town, South Africa; 3 School of Public Health, University of the Western Cape, Bellville, South Africa; 4 School of Nursing and Public Health, University of KwaZulu-Natal, Durban, South Africa; 5 Public Health, Societies and Belonging, Human Sciences Research Council, Pretoria, South Africa; 6 School of Public Health and Family Medicine, University of Cape Town, Cape Town, South Africa

**Keywords:** Barriers, Enablers, HIV treatment, PLHIV, South Africa, Universal test and treat

## Abstract

Ending AIDS by 2030 would depend on how successful health systems are in linking people living with HIV (PLHIV) into care. The World Health Organization recommended the ‘Universal Test and Treat’ (UTT) strategy – initiating all individuals testing positive on antiretroviral therapy (ART) irrespective of their CD4 count and clinical staging. This study aimed to explore the enablers and barriers to linkage to HIV care among adults with a new HIV diagnosis in a high-HIV prevalent rural district in South Africa. A qualitative study was undertaken to explore patients’ perceptions of enablers and barriers of linkage-to-care, using a life-story narration and dialogue approach. In-depth interviews were conducted with 38 HIV-positive participants sampled from a cohort of 1194 HIV-positive patients recruited from December 2017 to June 2018. Participants were selected based on whether they had been linked to care or not within 3 months of positive HIV diagnosis. Interviews were thematically analysed using a general inductive approach. Of the 38 participants, 22 (58%) linked to care within three months of HIV-positive diagnosis. Factors that facilitated or inhibited linkage-to-care were found at individual, family, community, as well as health systems levels. Enablers included a positive HIV testing experience, and assistance from the fieldwork team. Support from family, and friends, as well as prior community-based education about HIV and ART were also noted. Individual factors such as acceptance of HIV status, previous exposure to PLHIV, and fear of HIV progressing, were identified. Barriers to linkage included, denial of HIV status, dislike of taking pills, and preference for alternative medicine. Negative experiences with counselling and health systems inefficiency were also noted as barriers. Perceived stigma and socio-economic factors, such as lack of food or money to visit the clinic were other barriers. Community-based and health system-level interventions would need to focus on clinic readiness in providing patients with necessary and effective health services such as proper and adequate counselling. This could increase the number of patients who link to care. Finally, interventions to improve linkage-to-care should consider a holistic approach, including training healthcare providers, community outreach and the provision of psychological, social, and financial support.

## Background

South Africa has an estimated 7.8 million people living with HIV (PLHIV) in 2020 [1] and runs the largest public antiretroviral therapy (ART) programme in the world [2]. Although ART does not cure HIV/AIDS it has become more effective, well tolerated and even less complex, which enables PLHIV to adhere well to treatment which leads to viral suppression [3, 4]. In May 2016, South Africa became one of the first countries to roll out Universal Test and Treat (UTT) [5, 6], a strategy that was geared towards achieving the UNAIDS 2030 targets including the 95-95-95 goal, to have 95% of all people living with HIV knowing their HIV status, 95% of all people with diagnosed HIV infection receiving treatment and 95% of all people receiving treatment showing viral suppression [7]. UTT is a programme that is geared towards HIV testing and immediate treatment initiation regardless of CD4 count and WHO clinical stage [8].

One of the keys to the success of UTT is the implementation of interventions for linkage to care [9]. Other authors emphasize that timely and effective linkage to care and treatment has been identified as key to improved health outcomes [10, 11]. Linkage to care is defined as the successful completion of a first medical clinic visit within three months of HIV-positive diagnosis as evidenced by a record in Tier.Net [12]. The goal of linkage to care is to immediately initiate ART treatment after HIV diagnosis [13, 14]. Despite the implementation of the UTT strategy in South Africa, many PLHIV are yet to be linked to care. Studies have explored enablers and barriers related to linkage to HIV care within three months [15, 16]. A summary of these factors is included in Govindasamy and co-authors’ systematic review which revealed the most cited barriers to linkage to care were transport costs and long distances from home to the nearest health facility [17]. The problem of transport costs was further revealed in Sanga’s study [18], whereby longer distances from treatment sites appear to negatively influence linkage to care.

Other barriers mentioned by Govindasamy et al, including stigma, fear of disclosure of HIV status, staff shortages, long waiting times, fear of drug side effects, male sex, younger age, and the need to take time off work [17]. Larger household sizes, informal housing, and neighbourhood have also been identified as issues for poor linkage to care [19, 20]. In terms of socio-economic factors, some studies point to an association between being employed and being less likely to be linked to care, perhaps due to the difficulties of accessing healthcare services after working hours. Some studies point to higher education being associated with better care [17, 20]. It is critical that individuals with an HIV-positive diagnosis are rapidly linked to HIV care for better health outcomes [13, 21, 22]. However, individual, societal, and systematic factors continue to undermine efforts to link newly diagnosed HIV patients to care in most settings. While the barriers and enablers of linkage to HIV care pre-UTT are well documented, little is known about these factors in the UTT era, especially in predominantly rural settings with high HIV prevalence. In addition, there are very few studies that have classified these factors using an adapted version of the framework for a systems approach to healthcare delivery (FSAHD) [23]. This paper, which is a sequel to an earlier report on enhancing linkage to care in the uThukela district [24, 25], provides a qualitative analysis of a mixed-method study, on barriers and enablers related to linkage to HIV care in a high HIV-prevalent rural setting, in KwaZulu Natal, South Africa. It also provides some perspectives of PLHIV on the implementation of the UTT strategy.

## Methods

### Study design

A qualitative study design was used to understand the perceptions and experiences of patients on enablers and barriers of linkage to HIV care. This study was embedded within the main study [24, 25] and included in-depth interviews with newly diagnosed HIV-positive individuals. Linkage to care in this study refers to the proportion of the adult population (18 years and above) per facility who successfully completed a first medical clinic visit within three months of HIV-positive diagnosis and have been initiated onto antiretroviral therapy (ART) as evidenced by a TIER.Net record.

### Study setting

The study was conducted in the uThukela District Municipality (DM) in KwaZulu-Natal (KZN). uThukela DM is a predominantly rural district with a high HIV prevalence of 22.4% [26], compared to the South African HIV prevalence of 13.7% [27]. The district is comprised of three local municipalities (LMs) namely: Alfred Duma LM, Inkosi Langalibalele LM, and Okhahlamba LM and shares its western border with the country of Lesotho. Most HIV services in the district are offered to patients through the primary health care (PHC) setting. Participants included in this study were purposively selected from 18 public sector healthcare facilities based on the HIV testing uptake rates (Table 1).

**Table 1:** Selected facilities included in the Linkage to Care study in the uThukela district between December 2017-July 2018

	**Local Municipality**	**Name of facility**	**Type of facility/service**
**1.**	Alfred Duma	Ladysmith Gateway Clinic	Gateway Clinic
**2.**		Limehill Clinic	Clinic
**3.**		Outer West Mobile 1	Mobile
**4.**		Sigweje Clinic	Clinic
**5.**		St Chads CHC	CHC
**6.**		Walton Clinic	Clinic
**7.**		Steadville Clinic^a^	Clinic
**8.**		Watersmeet Clinic^a^	Clinic
**9.**	Okhahlamba	Bergville Clinic	Clinic
**10.**		Bergville Mobile 2	Mobile
**11.**		Bergville Mobile 3	Mobile
**12.**		Emmaus Gateway Clinic	Gateway Clinic
**13.**		Emmaus Hospital^a^	OPD
**14.**	Inkosi Langalibalele	Connor Street Clinic	Clinic
**15.**		Estcourt Gateway Clinic	Gateway Clinic
**16.**		Injisuthi Clinic	Clinic
**17.**		Ntabamhlophe Clinic	Clinic
**18.**		Wembezi Clinic	Clinic

^a^Facilities that were not part of the original sample but added later to replace facilities with low enrolment rates

**Community Health centre (CHC**)**:** A facility that normally provides PHC services, 24-hour maternity, accident and emergency services and beds where health care users can be observed for a maximum of 48 hours, and which normally has a procedure room but not an operating theatre

**Clinic:** A facility at and from which a range of primary health care services is provided and that is normally open eight or more hours a day based on the need of the community to be served

**Gateway clinic:** A primary health care (PHC) clinic, located in community health centre (CHC) or a hospital Out-patient department (OPD) where patients with minor ailments are seen by trained primary health care workers free of charge, before being referred to the CHC or hospital. Every CHC/ hospital has a gateway clinic directly attached to it to serve the people in the immediate vicinity of the CHC

### Sampling

Forty participants were randomly selected from a cohort of 1,194 HIV-positive patients recruited in the main study [24, 25]. Based on the outcome of the 4-month follow-up survey, we randomly selected 20 participants who successfully linked to care within three months of positive HIV diagnosis, and 20 who did not. A maximum variation purposive sampling technique was applied to include a varied representation of participants in terms of linkage status, age, gender, marital status, employment status, geographic area and location of health facilities utilized.

### Study tools

**Fig. 1:**
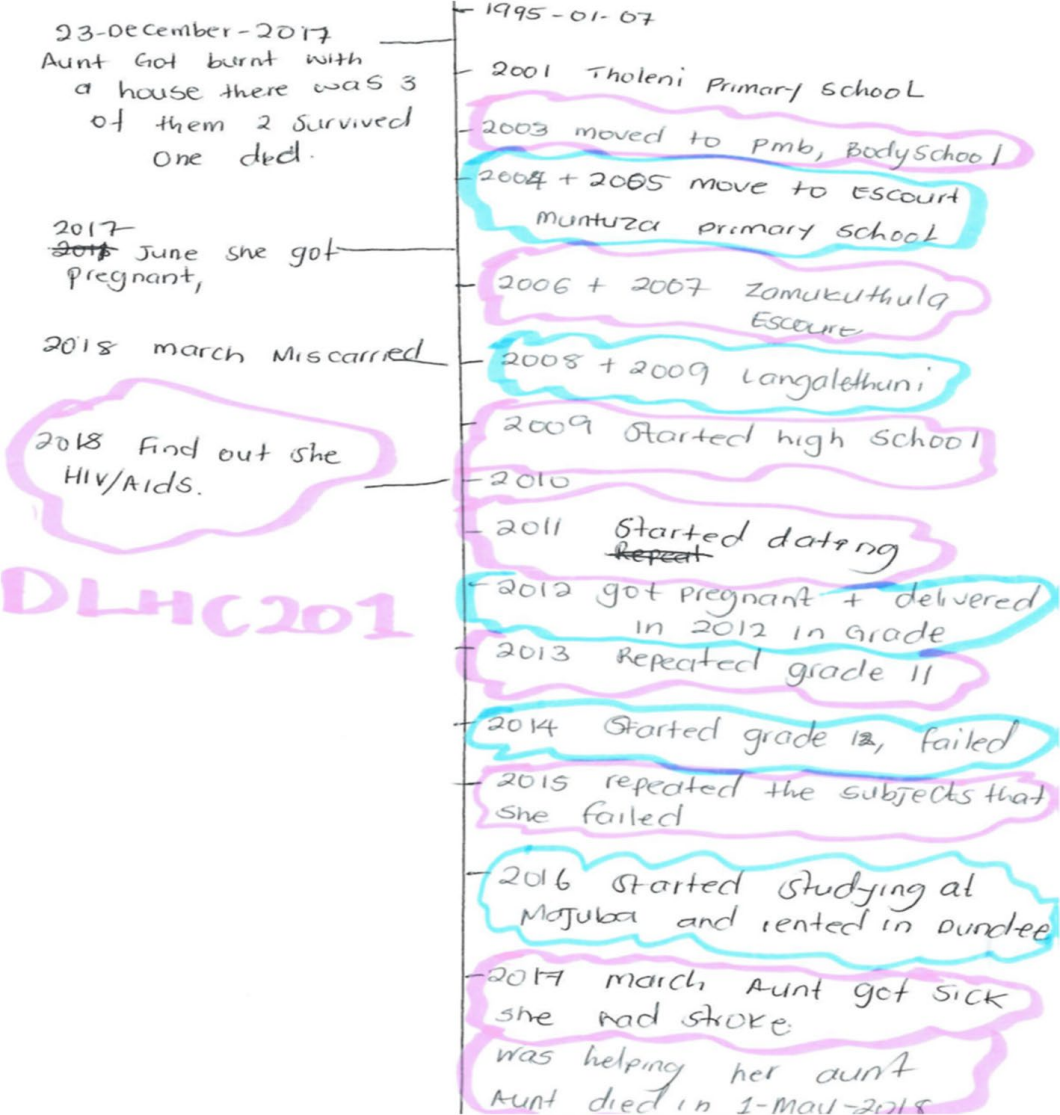
Qualitative tools - The life story exercise

A qualitative narrative interview guide, developed by experienced senior investigators and a life story exercise (Fig. 1), were used to collect data from the two subgroups of participating patients. The guide, which was used to explore five main topics: experience with HIV testing; disclosure; treatment uptake and adherence, internal and external stigma; and HIV support and care, was translated to IsiZulu by interviewers fluent in IsiZulu; and piloted in one local study site.

### Data collection procedure

Key informant interviews (KII) and life story narrations (Fig. 1) were mostly conducted in IsiZulu and based on participants’ availability in the place of their preference, to accommodate those who might not have disclosed their HIV-positive status. The life-story illustrations, used to triangulate interview data, involved a participant-led drawing of significant events that happened in the participant’s life from birth to discovering their HIV status. This exercise was designed to elicit information from participants using a paper to capture memories, which made the interview a collective process, allowing the participant to take ownership of the process, which lasted between 20 and 105 minutes, and was tape-recorded.

Ongoing review of interviews, through listening to audio, was conducted along with the debriefing of interviewers. This assisted with maintaining the quality of interviews and amendments to the tools and interview procedure. All KIIs were transcribed verbatim and translated into English. Quality checks were performed on all transcribed interviews by a qualitative researcher who is competent in English and IsiZulu languages.

Each respondent had a unique code, e.g., BISC_L_M47, added at the end of each quote to provide context. The first initial letters describe the facility area and the type (i.e., primary clinic, mobile clinic etc.), the ‘L’ describes linked participants, while the ‘NL’ describes those who did not link. This is followed by participants’ gender (F/M) and age at the time of the interview.

### Data analysis

A thematic content analysis driven by the main questions of interest was used to analyse the data sets. The analysis team, which comprised of three members, independently read through all the transcripts to gain a general understanding of the content and scope, and discussed initial emerging themes used to develop a structured coding framework. Coded information was linked to broader emerging themes across interviews. Data from the IDIs were coded independently using ATLAS.ti v8 software. Comparative analysis between two skilled qualitative researchers was performed to ensure analysis accuracy. The analysis process was informed by the interview questions, literature, and the inductive approach to the data [28].

## Results

### Demographic characteristics

Thirty-eight (38) participants, out of 40 were interviewed between October 2018 and February 2019, and two participants could not be reached. No efforts were made to replace them as data saturation was reached. Of the 38 participants, 22 (58%) linked to care within three months of HIV positive diagnosis.

Table 2, which illustrates the demographic characteristics of participants recruited from all 18 facilities, shows that the participants were all Black Africans, and the majority (24, 63%) came from clinics, while four (11%) were recruited from hospitals. This allowed for varying context-based experiences. Twenty-nine (76%) were female, and 35 (92%) were in some form of a relationship (married, cohabitating, or dating) at the time of data collection. Half of the participants had completed Grade 12, and about two-thirds (61%) were unemployed.

**Table 2:** Demographic characteristics of participants disaggregated by linkage status

**CHARACTERISTICS**	**LINKED** **n (%); *N*=22**	**NOT LINKED** **n (%); *N*=16**	**TOTAL** **n (%); *N*=38**
**Facility type**			
Clinic	13 (59.2)	11 (68.8)	24 (63.2)
Gateway	3 (13.6)	4 (25.0)	7 (18.4)
Hospital	3 (13.6)	1 (6.2)	4 (10.5)
Mobile clinic	3 (13.6)	0 (0.0)	3 (7.9)
**AGE** in years, median (IQR)	32 (24-36)	31 (22-32)	31.5 (22-36)
**Ethnicity**			
Black African	22 (57.9)	16 (42.1)	38 (100.0)
Colored/Mixed ancestry	0 (0.0)	0 (0.0)	0 (0.0)
White	0 (0.0)	0 (0.0)	0 (0.0)
Indian/Asian	0 (0.0)	0 (0.0)	0 (0.0)
**Sex**			
Male	6 (27.2)	3 (18.8)	9 (23.7)
Female	16 (72.8)	13 (81.2)	29 (76.3)
**Marital status**			
Married	2 (9.1)	1 (6.2)	3 (7.9)
Cohabitating	8 (36.4)	2 (12.5)	10 (26.3)
Dating	11 (50.0)	11 (68.8)	22 (57.9)
Single	1 (4.5)	2 (12.5)	3 (7.9)
**Educational level**			
Primary school/Grade 1-7	2 (9.1)	0 (0.0)	2 (5.2)
Secondary school/Grade 8-11	9 (40.9)	8 (50.0)	17 (44.7)
Matriculated/Grade 12	11 (50.0)	8 (50.0)	19 (50)
Post matric	0 (0.0)	0 (0.0)	0 (0)
**Employment in the past 3 months**			
Employed	10 (45.5)	5 (31.3)	15 (39.5)
Unemployed	12 (54.5)	11 (68.8)	23(60.5)
**Access to R200 (US$15) in case of an emergency**			
Yes	7 (31.8)	7 (43.8)	14 (36.8)
No	15 (68.2)	9 (56.2)	24 (63.2)
**Received earnings in the past 4 weeks**			
Yes	8 (36.4)	5 (31.3)	13 (34.2)
No	14 (63.6)	11 (68.7)	25 (65.8)

#### Perceptions on enablers and barriers of linkage to HIV care

Several themes and subthemes relating to patients’ perceptions of HIV testing and linkage to HIV care were identified. Factors that facilitated or inhibited linkage to care were found at individual, family, work, community, as well as health systems levels. Table 3 presents a summary of the general themes and subthemes that emerged from the data, which are categorized under three levels according to the adapted framework for a systems approach to healthcare delivery (FSAHD) [23] i.e., individual (service users), family/work/community (care team), and health system (organization) factors. These subthemes range from experience with HIV testing; disclosure; treatment uptake and adherence, internal and external stigma; and HIV support and care. We present in detail with illustrative quotes these themes and subthemes.

**Table 3:** Themes and subthemes on linkage to HIV care by levels - individual, family/work/community, as well as health systems levels

**Individual factors**	**Family/work/community factors**	**Health system factors**
**1. Reasons for seeking HIV testing**		
• Routine check-up • HIV related symptoms • Feeling sick • Prenatal care	**•** Spouse-related elements	
**2. Barriers to linkage to HIV care**		
• Denialism and lack of emotional readiness • Preference for alternative medication • Dislike for pills • Progression of HIV • Experience of side effects from ARVs • Fear of non-adherence • Perceived stigma	**•** Socio-economic factors (lack of income, distance to clinic, lack of transport/food, inability to get time off from work) **•** Lack of disclosure to partner or family members	**•** Poor counselling and clinical procedures **•** Health systems inefficiency
**3. Enablers to linkage to HIV care**		
• Fear of HIV progressing • Acceptability of status • Disclosure • Previous exposure to PLHIV	**•** Support from family and friends **•** Support from work/peers **•** Prior education on ART	**•** Positive testing experience **•** Accessibility to ARVs **•** Change in the treatment guidelines. **•** Linkage to care study impact
**4. Perceptions on the implementation of UTT**		
• Happy with the implementation of UTT • Opportunity to continue with life • Non-acceptance of the implementation of UTT	**•** Family/friend bad experiences with old HIV treatment guidelines	

### Reasons for seeking HIV testing

Participants expressed different reasons for HIV testing. Some reported they tested because they wanted to know their status, others tested because they fell ill, while some patients reported going to the clinic because they noticed that they had developed HIV-related symptoms. One of the HIV-related symptoms that was mostly reported by the patients is loss of weight. Other patients had HIV testing as part of prenatal care. Spouse/partner-related factors were expressed as some of the reasons to go for HIV-testing. These included partner promiscuity, partner’s disclosure of HIV-positive status, partner being sick, and routine couple check-ups.

***Routine check-up***

“*I came here to the clinic…, I just told them that I want to do all tests. They did all tests. BP, diabetes, HIV, and HB blood…” ****(KEMG_NL_F29)***

***HIV related symptoms***

*“Ai, I saw that I was not feeling well, and I was losing weight. I decided to go and check my status.”* ***(KEMG_NL_F29)***

*“In 2017 November, I started losing weight, … My mother said, I must go to the clinic; I went … and got tested. They told me at the clinic that I am positive, I was shocked.”* ***(EOWM_L_F21)***

***Feeling sick***

*“I had a sore throat, then I went to the clinic and then they said I must first test….”* ***(BNHC_NL_M20)***

*“It’s because I was not feeling okay, and …, I was suspecting that I am not well because I was always sick. I then decided to go to the clinic, and I then tested.”* ***(UCSC_NL_F37)***

***Prenatal care***

*“It’s because I was pregnant. I was eight months [pregnant] because I had checked the time I started to come to the clinic and they said I should come back when I start with the antenatal clinic, I went back.”* ***(DLHC_L_F23)***

*“I tested because I was pregnant. It was…. Everyone who was pregnant …. What can I say? You were forced to do an HIV test if you are pregnant.”* ***(ESVC_L_F31)***

*“After one week I went to the clinic and said I was pregnant. I then requested to test. Yes, the results were positive.”* ***(KB2M_L_25)***

***Spouse-related elements***

*“He was sick, he was very sick and then I suspected that I also have it [HIV] because he is someone who likes women. So, he started getting sick as someone who had flu, he had endless flu, …. He came and tested, and the following month I came to test, and I found out that we were both sick [HIV positive].”* ***(DIGC_L_F25)***

*“We just decided to go and check because he was insisting that we go and test so that we know our statuses.”* ***(BISC_L_F23)***

*“Yes, it is because I know I had already slept with this person. Whereas, I had observed his behaviour…. Because the first is that this person drinks, there are always women and all that. Oh, another thing I had an infection. The infection was on my private part, I had a discharge which had a bad smell and all that. Then I had to be treated for STI, after that I needed to know my status, you know. So, it happened like that.”* ***(EWCC_L_F24)***

### Barriers to linkage to HIV care

Barriers to linkage to HIV care were similar for all the participants who struggled to be linked to care within three months and those who did not link to care. These included an interplay of health system factors, family, work, community-level factors, and individual factors. For some, there were clear-cut issues, whilst for others it was a combination of different factors that led to non-linkage to care or discontinuation of the linkage process. Most participants who did not link to care did not disclose their status to significant others including their partners.

#### Individual factors - Barriers

Individual-related factors appeared to be the most frequent emerging barriers to linkage in care when compared to health and family/work/community level factors. They included denialism and lack of emotional readiness, dislike for pills, perceived negative side effects from ARVs, perceptions about ARVs, preference for alternative medications and perceived stigma.

##### Denialism

Denialism emerged as a barrier to linkage to care. Some participants mentioned the need for a second opinion to confirm their HIV status. One participant who had an experience relating to false positive results, was doubtful about her current positive status, and thus did not link to care. Denialism also appeared to be based on a lack of proper HIV knowledge such as how one gets infected, or the fact that one is not sick or has any HIV-related symptoms.

*“Sometimes it happens that when you test, they find that you are [HIV] positive, … when you go back again you will find that you are negative. ... Even if I find that I have it [HIV], it is me who needs to decide whether I will take treatment or what to do. I do not believe even now ai, a whole year has passed. Last year I thought maybe I would get sick; I am still like this… Because I also believe that I do not have it [HIV]. Ok, I tested but I did not test again.” (****EWCC_NL_F32)***

*“… HIV is better than diabetes, when I have accepted the situation, I will go … and check again if the results are still the same, ... I will plan to go when I am ready. I do not have a problem with pills, just that I have not accepted the situation.”***(*DLHC_NL_F24)***

##### Other modes of treatment used by participants

The idea of using other modes of treatment such as traditional (including immune boosters) or religion rather than starting HIV treatment came up as the reason for not immediately linking to care. Participants stated that they preferred using traditional medicine before going back to the clinic for HIV treatment. They also expressed their inclination to traditional-based remedies and how they used them as immune boosters.

*“I have tried that because I want to start treatment after I have taken traditional medication first. Do you understand me? I did it, I need to finish using these things [traditional medication] then go back to the doctor.” ****(UECG_NL_M40)***

*“Yes. What I used to take were these things [traditional medication], what do they call it, immune boosters, … I tried it soon after I discovered in 2004, 2005.” ****(UECG_NL_F37)***

*“Yes, I once tried Gambu (Traditional herbal medicine), I once bought Gambu and drank it. No, they do advertise on radios and say “Drink Gambu, it will assist with CD4 count and so on and so on.” ****(UCSC_NL_M38)***

Some participants prefer to rely solely on their religious beliefs to tackle medical issues. A participant explained the possibility of getting cured of HIV through prayers, beliefs and other religious rites, and thus did not require to be linked to care.

*“…I do not know whether it is miracle because sometimes it happens that they cannot see anything wrong at the hospital and it helps to brighten up things like that. Maybe if I can believe it can happen, let us put it like that. But they say if, let us say you have to undergo an operation, once you attend that church, given water, prayed for, because mostly it works with prayer, your problem becomes clear. So maybe it can happen that when I got tested, I was HIV [positive]… You will never know how miracles work you see." ****(UECG_NL_F37)***

##### Dislike for pills

Dislike for pills was not a common theme, however, it is assumed to have played a part in poor linkage to care as individuals who shared concerns with taking pills, did not link to care at all. One of the participants said:

*“The reason for not taking treatment is because even at a young age I used to break the pills into pieces and then drink it. So, I do not know, I need to get information on whether ARVs still work if you break them into pieces. I want to do research because I have a problem with the pills, they just stop in the throat. That is another reason why I am not taking ARVs because I have a problem swallowing pills. Secondly, I told myself because they say once you have started ARVs you have to continue with them for life. I have a problem taking a pill because even when the Doctor gives me medication, I hardly finish it. When I recover, I go on with life. Maybe those are the reasons that made me not take medication because even the Doctor’s medication…, we are human beings …, the pill is not nice. Even though it helps it will not be easy to take it for life. I do not criticize them because they are helpful … but I took that decision [not to link] based on these reasons.” ****(UECG_NL_F37)***

##### Progression of HIV infection

The issue of waiting for the HIV infection to progress before seeking care emerged as the reason for not linking to care. Some participants believe that there is no reason to seek care if they are not feeling sick. Participants expressed themselves as follows:

*“Another problem I have is, I do not know whether it is because I am still fine, I do not bother myself with other things like taking medication and so on... I did not bother to go there simply because when I left there they said since I have to start medication I must go to the nearby clinic. No, I never [went]. I just think because I am still all right, I do not want to take something that I will not use…” ****(UECG_NL_F37)***

*“So, it’s up to me whether I take that decision when I am not well then, I will go to the clinic to take medication. So, I told them that I am still okay and, I would come when I am ready. No, I realize that I cannot, I will go when I see that I am sick but for now I am still fine.” ****(BISC_L_F23)***

*“…I will start taking them [ARVs] when I am sick, because if I am saying that I am taking them while I am not sick, they will make me sick and how am I going to survive?” ****(UCSC_NL_M38)***

##### Perceived medication side effects

Some participants did not link or remain in care because of their perceptions and experience of the negative side effects of initiating ART. Participants who had not linked or retained in care expressed their fears regarding this:

*“Another reason [for not linking to care] is that sometimes I see people… I know. I do not know how they do this thing of disclosing that they have HIV. There is someone who has shown me HIV medication, he says “he is taking pills”. When I see him, he has changed, he is not like before. He changed when he started. Sometimes people gain weight and become fat while others… their skin becomes darker. I am scared that will happen to me when I start taking pills.” ****(BNHC_NL_M20)***

*“I told even XXXX [fieldworker] that …, they [ARVs] are causing sickness; really, … I would not continue with them [ARVs]…, because if I am saying that I am taking them while I am not sick, they will make me sick and how am I going to survive?” ****(UCSC_NL_M38)***

*“…plus, they [ARVs] are causing disability, I saw that in another lady.” ****(UECG_NL_M40)***

##### Fear of treatment non-adherence

Participants expressed fear of treatment non-adherence, based on information received during counselling that ARV is a life-long treatment. These are extracts from some participants who felt they might default in taking treatment if they start, hence they did not link to care.

*“It is very difficult, maybe I can take my treatment for two days. That is why I do not like taking it [ARVs] because they say if you have started you are not supposed to stop.”***(*BNHC_NL_M20)***

*“No, it is just a matter of managing time. I am someone who forgets, I am not used to taking pills. I will just forget that I must… but I will get used to it.” ****(ESVC_NL_F20)***

*“*…*Yes, because I do not want to default, I was told that I must not take them and then default. Another thing is [that] I do not want to waste state resources. I took a decision that I will accept and look after myself, that is why I am still alive today.”***(*UECG_NL_F37)***

##### Perceived stigma

For some participants, linking to care meant their HIV status would be known by other community members, as queues in the clinic segregate patients, based on the services they want to access. Some participants preferred linking to care only if the care is provided in their homes, so that they could have some privacy. Others felt they needed to attend clinics far from their community because they feared being stigmatized.

*“Here this person has TB, he/she is carrying a yellow card. When you check, the other one is carrying a white card and the other one is carrying a green card “we [PLHIV] are carrying green cards, …”, do you get that. Now it is seen that those who are carrying a green card, are those who are like [HIV-positive].” ****(UECG_NL_M40)***

*“Yes, including carrying these papers and cards, on that I can say that it discriminates the person. Some of them are even shy that they will carry these envelopes that are of this side. Because they say it is those who are collecting pills. It is written on that door “For those who are collecting pills, here is for those who are this”.***(*DLHC_NL_F24)***

*“… there is no one who knows me here. I come to the clinic to do what I need to do; it is not the same if I am around my area.” ****(UCSC_NL_F37)***

### Health system factors - Barriers

Health system barriers were the second most common barriers to linkage in care. In general, participants who expressed a negative testing experience did not link to care. They noted poor counselling quality, clinic procedures, and health system inefficiency as health systems-related barriers for not linking into care. Participants expressed that nobody asked about their well-being after initiating treatment.

#### Poor counselling quality and clinic procedures

The issue of counselling quality was explored when participants recalled their testing experience. Most participants in this study revealed that the counselling period was short. They also alluded that its emphasis is on ART education with little information on the virus, viral load, use of condoms during sex, etc., like in the case of the participant below:

*“...she [counsellor] said to me “let us check”, …“you know what these lines mean if they are two?”, I said “Yes”. She said to me “You are [HIV] positive”, I said “How come?”, she said “I do not know the things you are doing”… You know she was not patient, just to explain to me…. “No, you see the results, what do you want now?” She said, “My sister the results are like this nothing will change”. I said “all right”, …when I went outside, I was confused. I asked “How?”, she said “Take this file and go to the nurses they will explain to you”… I took my card and went out. The way I was hurt,… she was supposed to explain that it’s like this and this,...” ****(DIGC_NL_F36)***

Participants alluded to the issue of confidentiality in the counselling sessions with one participant suggesting that the perceived lack of confidentiality led her to not seek care even at a different facility:

*“I was going to go to LH because it’s not the same as here …. At LH, they can maintain confidentiality. Here [current facility], they cannot keep someone’s secret. Let me just keep quiet like that and say if I am ready, I will go. In actual fact, I want to understand [new status] and accept. I do not have a problem with treatment. HIV is better than diabetes, when I have accepted the situation, I will go to X and check again if the results are still the same. I do not have a problem with that; I will start treatment.” ****(DLHC_NL_F24)***

*“What also makes me not to collect from this clinic, which is closer to home my child, I will be open [up] as you have said that we must be open. In that clinic, I once told XXXX, that if workers see you enter the counsellor’s room or carrying a file because they [those collecting ARVs] sit alone, they will gossip about you which is wrong because in workshops that I am attending that [HIV positive status] is a secret”. ****(*UCSC_ *L_F54)***

#### Health system inefficiency

A few participants highlighted some instances of inefficiencies within the health system as hindrances to initiating HIV treatment, like the participant below:

*“What made me not to come back? I was irritated there. After checking [testing for HIV]... they said I must go and check for TB. …when I came back, they said they did not see the results, [that] I must do it [test] again. When I need to come here, it is far; I do not have money, and now I need to go back and check again. When I came to check [for] the results, they said I should come back another day and that there were no results again ...I waited for a long time and they could not get my file. They said I should start afresh and make a new file. What makes me angry are the things that I have been doing, checking and so on, when they said I should start afresh I did not have money to go back..., I did not go back. I decided that at least I should go to another clinic ... When I wanted my file, they said they did not see it, because it meant they do not pay attention. It is better if I go to another clinic.” ****(ECHC_NL_F34)***

### Family, community, and work level-factor - Barriers

Most participants who had issues with linking to care did not disclose their HIV status to significant others. The issue of food, loss of time at work, and lack of transport money are some of the subthemes that emerged at this level. One participant had concerns regarding losing time at work and telling his manager every month that he was going to the clinic.

#### Socio-economic factors

Lack of income appeared to be a factor in not linking to care. One participant who did not link into care at 3 months expressed the possibility of not having (enough) food at home as a hindering factor to linking into HIV care. This is based on the participant’s understanding that ART should not be taken on an empty stomach. Participants complained about not having money to pay for transport going to the clinic.

*“I wish to [take pills] but what can I say, I am not ok because what I am afraid of my sister, is to start while I am still unemployed. Then it happens that I have a problem with food and pills will make me sick... Yes, I fear that. Sometimes it happens that the food is not enough, and I prioritize the baby. If I start taking pills when the time is 9 to take pills, I do not have something to eat. How can I take a pill, and at the end I will be forced to quit? I do not have a problem with taking pills, that is what I fear.” ****(UCSC_NL_F25)***

*“It happens that I do not have money for transport.” ****(UCSC_NL_F25)***

*“… then I had that problem. You find that to my boss I will say I am going to the doctor, then they will say “Every month?”.***(*UECG_NL_M40)***

### Enablers to linkage to HIV care

Many of the PLHIV who tested positive and successfully linked to care identified several enabling factors. These factors are classified under individual, family, and health system factors (Table 3).

#### Individual factors - Enablers

##### Fear of HIV progressing

More than half of the participants who came to the facility did so when they were already ill. This was a huge motivator for people to link to care, with most stating that they feared HIV progressing and that they wanted to live longer. These participants expressed feelings of being scared to die or be seen as sick by other people, hence they linked to care.

*“I am scared to die; I am afraid that people will see when I am sick.”***(*DIGC_L_F25)***

*“I wanted to live and for you to mitigate it [HIV], you need to start taking medication early because if you are saying “I will see after this time”, you can end up suffering from another disease aside; and now you are very sick even medication can’t assist.” (****EOWM_L_M51****)*

*“I thought that since I know my status, why do I have to wait? And I need to avoid a stage whereby people will notice, and also my family and the entire community that I am very sick. It’s better to start treatment now, I wanted to be fine. Since I know, the intention was not just to know, I wanted to take action after knowing.”* (***EWCC_L_F24****)*

A participant who started same-day treatment did so to avoid a drop in his CD4 count and to prevent the illness from getting worse, as was the case with his sister.

*“I started on that day. That lady drew my blood, and it was not that bad because I had made a decision. I know that even if it [CD4 count] goes up today, you will never know what it will be like the following week. My sister had that problem. They stopped her… they stopped giving her medication because her CD4 count was high not knowing that it was going to drop. She almost died when they gave her medication. Also, days are not the same as one is living under such depressing conditions. That is why I told that lady to give me medication while I still felt fine.” ****(SEUWBC_L_M40)***

The desire to live longer and continue with life with family was one of the enablers of linkage to care that emerged from the data.

*“It’s that I want to live with my children and mother. I still want to live.” (****ESVC_L_F31****)*

*“Mmmh! I think it’s about being ready and telling yourself that it’s about life, I just want to live. To make sure that I do not have problems so that I can look after my children because dying now while they are still [young]. What encourages me is that there are people I am living for.” (****EWCC_L_F30****)*

##### Acceptance of status

Accepting one’s HIV-positive status compared to denialism was another individual enabler for participants who linked to care. This is because those who accepted their status were receptive to the advice given by Health care providers about the importance of early initiation to treatment.

*“Eh, the time I was here…eh I started going to the clinic; I was satisfied because I was assisted. I accepted all that they were saying. I had no choice. There was no other decision I could have taken because I cannot kill myself. I decided to take a decision that I must use what they are going to give me. I took that decision [accepted to link to care].” ****(ECHC_L_M41)***

*“I am just free; I have indeed accepted. As I am saying people who know about my situation are supportive, they look after me, they ask me whether I have taken medication and other things. So, I realized that these people have accepted me in all forms, there is no reason to distance myself from them because they are bringing me close to them as a human being.”* (***EOWM_L_M51****)*

##### Disclosure and family support

The narrative suggests that the majority of the participants who had been linked to care in this study disclosed their status to their families and friends and were not stigmatised. They claimed that the disclosure enabled them to initiate treatment, through the support provided to them such as emotional care, to ensure they adjust to their new status.

*“I told my sister; she knew our mother’s [HIV] status because I was staying with her. I told her; she did not have any problem. She said to me “Z you must take pills, do not stop taking them and if they are finished, you collect them.” ****(SDKBVC_L_F27)***

*“Oh, eh I told them at home so that they can support me and remind me every day if I have forgotten to take medication. As I have said if I am busy, my grandfather collects it for me. If I did not tell grandfather, if I sent him to collect this medication he was going to say, “I am going to do what, where?” ****(UCSC_L_F54)***

##### Prior exposure to ill PLHIV

Having prior exposure to PLHIV also motivated some individuals to immediately link to care after discovering their status, due to the fear of the disease progression and becoming more debilitating. A participant explained this in the excerpt below:

*“… When she [Friend] found out that she is [HIV] positive, … She said, she will not be able to take pills. I said, ‘I will not push you to take pills because a person must do something, he/she loves’. We went back home, you know she stayed a long time after a year she started getting ill. When she was sick, it was very bad. She had things on her body, spots, and even her face and hair changed. From there I decided that when I find out that I am positive I will not make the same mistake. I will start treatment immediately.” ****(ECHC_L_25)***

### Family, friends, and community level factors - Enablers

Those who disclosed their status were more likely to receive support from the community, family, and workplace. These sources of support are discussed separately below.

#### Support from family

Participants claim that families played an important supportive role in enabling them to initiate treatment. An example of such support is demonstrated by this extract below:

*“I told them that the results I received indicated that, I am positive. They understood and said they will support me; I must continue going to the hospital.” ****(DIGC_L_F35)***

The data showed that exposure to family members who are HIV-positive was a crucial factor for participants in this study to link to care, since these family members understood their situation and provided emotional support and care that encouraged participants to accept their new status:

*“I ended up explaining to her and it was just that she is someone who is very open… She then told me ‘I started a long time; I am living with this situation; I do not have a problem. I am still alive…’ She told me that she started [treatment] a long time ago.” ****(BISC_L_M47)***

*“It helped so much [to disclose], even though I have not told all the people who are close to me because people are not the same. But it helped me because I feel like someone who is accepted and supported all the time. Again, people I have told, ask me all the time whether I have done certain things “yes I have done it”, “Did you eat?”, “Yes, I have eaten”, “are you having enough sleep?”, “Yes I sleep enough time”. They also say that I must make sure on that one because I will turn back my life.” ****(EOWM_L_M51)***

#### Support from peers

Of the few participants who were interviewed in this study, only one disclosed their status to their work colleagues, and these were also supportive.

*“I told her that I have tested, and they found that I have HIV. Ah she accepted and became supportive as I was saying that she is very supportive. Eh at work…ah what can I say… there is a lady who is my friend. Ah I told her that I tested, and I discovered that I have HIV My friend is also very supportive because when I am at work, she helps me so much, she would say ‘my friend since it has been found that you have HIV, you need to eat fruit and vegetables, ensure that you eat healthy food and make sure that you take your medication on time’. She was someone who was encouraging me most of the time because she is someone who I was very close with, even now we support each other.” ****(EOWM_L_M51)***

#### Prior education on ART at the community level

Prior education at the community level about ART enabled participants to link to care.

*“As I have said, there are workshops that we are attending since I am on a community committee. They gave me the information; do you see that? They explained that ‘if you start early, you are helped easily and become healthy more than anyone.’ I said, ‘I have found a jackpot if it’s like this’ …To me it was a mixture of that information, I said ‘This is the big opportunity let me accept it with both hands.” ****(UCSC_L_F54)***

### Health system level factors - Enablers

The health system factors that enabled people to link to care included: positive testing experience; the availability of ARVs; being transferred to a closer facility; change in the treatment guidelines; as well as presence of the Linkage to care research team in the facilities.

#### Positive testing experience

Positive testing experience of getting adequate counselling, patience, and emotional support from healthcare professionals enabled most participants to link to care:

“*They treated me well, with respect, they tested us. The first time they said, ‘There is no virus’, ... When I came back for the second time, they told me that ‘you see you have HIV now’, they said ‘What do you think you should do, do you want to start taking pills or not?’. I said, ‘If you have it there, then it must be something I need to use’, then they registered me, and I entered another door for collecting pills. They said I must go for…taking blood.” ****(BISC_L_M47)***

*“They treated me well; I was indeed treated well because they took care of me, and they taught me that HIV does not kill. I went back to having that confidence [that] even though I had seen that I am sick, I did not feel bad that much. But I felt that I am sick, but they comforted me.” ****(DIGC_L_F25)***

*“No, I have never… I am treated well I have never had a problem. I would be lying; I have never met someone who speaks disrespectfully. Because even on this side when I started, they were fine, they even said that ‘if you have a problem, if there is something that is worrying you just say it, if you are sick and there is something you do not understand just say it.’…”***(*ECHC_L_F25****)*

#### Accessibility to ARVs

Most participants claimed that healthcare providers (HCPs) were able to assist those who had difficulties accessing ART in the facilities they tested in with transfers, which aided with linkage to care:

*“They did not give me medication, but they wrote a letter for me, I said I do not have money for transport, it is better if I go to the clinic that is not far from me. It will be difficult because I would have to walk that mountain if I did not have money. Sometimes I used that bush route, where you cross the bridge, I walked to get medication.” ****(ECHC_L_M41)***

#### Change in the treatment guidelines

Participants highlighted that the change in the HIV guidelines to UTT and being able to immediately start treatment motivated some participants to link to care.

*“What gave me hope is that you do not wait to test how much your CD4 count has decreased. When you have found it [HIV-positive], you just start medication. That gave me so much hope, I did not have any problem because they said, ‘You will take your pills now’, … When I came back to take medication for Shingles, I told them that “Eh I have been helped” …I can say that through testing I became aware [of status] and that helped me. I have started medication and I have seen the difference and finding this [testing] really helped me I would have been injured. My body recovered very early.” ****(UCSC_L_F54)***

Another participant who has been living with HIV but did not qualify to start with treatment under the previous guideline mentioned how UTT enabled her to immediately initiate ART as soon as she tested again, after the introduction of this guide in health facilities.

*“Before, you were testing if your CD4 count is still high, you were not allowed to take medication. It was just that and I was well all this time until last year when I observed that there is a change in my body. I went back to the hospital and tested again, then I started medication.” ****(ESCV_L_F31)***

#### Impact of the linkage to care study

During the administration of the survey by the Linkage to care study team, participants got to briefly engage with the team and were exposed to some information about what it means to be HIV-positive. For one participant, this aided with linking into care, as expressed below:

*“Before I entered when the nurse gave me directions, it started from XXXX [team member] who prepared me psychologically. Because he was also asking me whether I think pills help? What are the advantages and disadvantages I have seen about pills? Then I told him even though I was laughing at some point because I love to laugh before my child, we were sacred of these pills.” ****(UCSC_L_F54)***

### Patients’ recommendations

During the interviews, a small number of participants suggested strategies that could be helpful with linking and staying in care. Table 4 highlights these strategies which are categorized by themes accompanied by some extracts from the data.

**Table 4:** Recommendations by patients

**Recommendations**	**Extracts**
Availability of mobile clinics	“Oh, I wish that people with HIV…. sometimes it happens that maybe someone who is infected is unable to go to the clinic. Maybe mobile clinics can assist in those areas so that they can get medication and be ok.”
Creation of support groups	“What I can say, you see we can be a group where we disclose, sit with others and make people aware. We must tell them, we must not be afraid that “as you can see, I have this HIV, do not be afraid since I am telling you that I have this disease”. I can advise that “if you do not have it, you must know that you must protect yourself and also continuously check your status”. You must know if you are still fine, so that you continue having a good life."
Faster administration	“Yes, sometimes I see that in the clinic there are people who have cards that are written Department of Health. When they are at the clinic, they just register and take their medication maybe two or three inside then they go. It would be better that way.”

### Implementation of Universal Test and Treat (UTT)

#### Patients’ perceptions of UTT

During data collection, participants got an opportunity to express their views on the implementation of UTT. These views varied, with most participants perceiving UTT as helpful, while some felt it was a rushed process that required more time to implement.

#### Happy with the implementation of UTT

Many of the patients expressed general feelings of happiness on the implementation of UTT. The happiness was based on the realization of getting immediate treatment and that they would not have to wait till they fell ill.

*“Ah, I am fine; I am happy about it. It helped me because she said that I shouldn’t wait to after some time. I must start now.” ****(EOWM_L_M51)***

*“I was happy because when I arrived, they told me that when you test, and if you have it [HIV], you start them [treatment]. I did not know, I thought you start by learning…” ****(SDKBVC_L_F27)***

*“It made me happy because I am scared to lay down and be unable to wake up, I was very happy. I felt very happy.” ****(DIGC_L_F25)***

#### Opportunity to continue with life

Others saw the implementation of UTT as a better intervention as it gives them a good chance at quality of life compared to the old HIV treatment guidelines.

*“Because I do not see the need that comes today, and you take them [treatment] another time. They are doing good in clinics because before I heard that they were taking your blood first, then you will come back when they see the state of your CD4 count and take pills. It’s fine now because when you see that you are positive, you start pills and continue with life.” ****(DLHC_L_F23)***

*“It [Test and treat] helps, it was helpful to me. I think that system [UTT]… remember that before I know that you needed to wait for your CD4 count, they take your blood and all that. So, it is… I think this new system of starting medication helps very much. You see that you are able to suppress it [HIV] at an early stage, without it reaching stage 1 or 2. So, it was helpful to me because I have never gotten sick, and even now I do not have any problem.” ****(DLHC_L_F230)***

#### Family/friends bad experiences with old HIV treatment guidelines

Other participants expressed their acceptability of the implementation of UTT based on bad experiences by a family member or friend who was diagnosed during the old HIV treatment guidelines.

*“He [friend] got tested and they did not put him on treatment. They said the CD4 count, I cannot remember whether it was 600 or what. They said it is high. His wife gave birth to twins, and they passed away. He was also involved in a car accident. After that, his CD4 count dropped drastically. He also had depression after his mother passed away.”***(*SEUWBC_L_M40)***

*“I was happy because I thought about my brother, it was the time whereby you start by treating TB before you start HIV treatment, then you fall sick and die. It is very good to test and get medication without starting by treating TB.” ****(SDKBVC_L_F37)***

#### Non-acceptance of the implementation of UTT

While many participants accepted the implementation of UTT, a few were not comfortable with the idea. The view from these participants was generally to wait for the CD4 count to decrease or wait for disease progression.

*“[Test and treat] even though you have not accepted the situation? I can say that it is not helpful but when you are taking them [HIV test] constantly, you get used to them [HIV result]. Then you tell your heart that because you tested and found out that you have it [HIV]. But you feel that you can wait a bit and then come back after some time. Alternatively, you can test again and when they say that you have it [HIV], just say ‘Okay, please give me another date so that I will come back again’.” ****(BISC_L_M47)***

*“Ah, I think it is the correct thing to wait for the CD4 count to decrease. I have seen from other people that it is not easy because when you start treatment there is that thing, the body is scared to start a new thing. So, it is better if I am still myself.”***(*EWCC_L_F30)***

## Discussion

This study aimed to investigate enablers and barriers to linkage to HIV care among adults with a new HIV diagnosis in the uThukela district in KZN. The majority of the 38 interviewed participants consisted of young people aged 23-36 years, who were at school, employed or looking for employment, and unmarried at the time of data collection. Males were underrepresented in this study, at 24% (9/38), because fewer men compared to women were recruited in the main study [24, 25]. As reported in the main study, accessing men via the health facility continues to be a challenge, as men do not visit the health facilities as frequently compared to women [29–31]. However, the few men who participated provided important insight into some of the challenges faced by men when accessing HIV care; this will require further investigation with a larger sample.

The “One Man Can”, which is a rights-based gender equality and health programme intervention, and the Decentralized Medication Delivery (DMD) are proven interventions that have successfully reduced masculinity-related barriers in accessing HIV services [32]. Evidence from South Africa shows the potential these service delivery models have in increasing retention in care and medication adherence among men living with HIV [33]. The MINA and Coach Mpilo campaigns, which provide men with information and support to get tested for HIV, initiate ART, and remain in care, are other interventions that have been implemented in South Africa [34, 35].

Adherence clubs or support groups, in which patients gather in small groups outside of regular clinic lines to pick up their pre-packaged medication and discuss adherence, play a key role in improving HIV linkage and retention rates. Adherence clubs and support group initiatives also help decongest clinics and reduce patient discomfort. For example, as part of a package of services that includes counselling and peer support, patients who are clinically stable on treatment and have demonstrated good adherence can be offered a repeat prescription collection strategy that allows them to pick up their medication in a less time-consuming manner than general clinic queues [33]. In a study conducted in South Africa, the use of adherence clubs led to better participant retention in care after a year, as well as viral suppression, particularly among men [33].

Of the 38 participants, 22 (58%) linked into care within three months of testing, as per WHO’s definition of linkage. The outcomes of this study show that linkage to HIV care is influenced by an array of individual, health system, and family factors. The study was also able to show both positive and negative views on the implementation of UTT from patients. The outcomes are condensed into five themes and discussed below:

### HIV testing, perceptions on risk of HIV infection, and physical health

HIV testing identifies those unaware of their HIV status and needing linkage to care and treatment, a crucial step for prevention. A significant finding and concern in this study is that some people only visited the clinic to test for HIV when they were already ill. This is consistent with other studies that revealed that deteriorating health and being symptomatic are factors positively associated with HIV testing and therefore linkage to care [36]. Besides being sick, the prospects of being sick or negatively affected by the disease in the future motivated individuals in this study to link to care. Also, HIV risk perception arising from individual’s knowledge of their own and their partner’s sexual behaviour, was a motivating factor for others to link to care. Loggernberg et al, found in another KZN study that participants did not want their physical bodies to transform because of a progressing HIV disease, hence they linked to care on the same day [37]. The high success rate in linking to care for some of the participants had been the experience of deaths and sickness of significant others, where treatment was not sought. It is commendable though that some of the participants went for HIV testing as part of their routine health check-ups, prenatal care, and couple health status check-ups.

Although participants’ heightened risk of HIV infection, and deteriorating health enabled them to link to care, there is also a disjunction between perceived risk of HIV infection and perceived physical health. Some of the participants did not believe their HIV-positive results based on their known sexual risk behaviors, and hence did not link to care, while others did not link to care due to their perceived physical health. In addition, individual barriers such as fear of changing their physical appearance, influenced linkage to HIV care in this setting. Some of the young participants reported not having initiated ART due to fear of looking sickly because of the medication, while others ensured that they started ART to maintain their ‘healthy-looking’ appearance. This disconnect calls for more HIV health education programs in the communities as well as in the facilities, emphasizing HIV transmission and the benefits of HIV treatment. For example, in 2016, the Prevention Access Campaign launched the "U = U" (Undetectable = Untransmutable) campaign which aimed to spread awareness that HIV-positive individuals who are virally suppressed and consistent on ART cannot transmit HIV to sexual partner(s) or through giving birth, a notion which has been publicly endorsed by the World Health Organization, U.S. Centers for Disease Control, and National Institutes of Health leadership [38]. Suggestions have also been made for U=U to be integrated into HIV counselling [38]. Information on U=U has been emphasized to have preventative benefits, due to its potential to reduce HIV stigma and improve uptake of HIV testing and treatment, resulting in improved health outcomes for PLHIV and lower risk of transmission to others [39, 40].

### Health system inefficiency (Falling through the cracks)

Participants’ perceptions of the poor quality of health services, characterized by the inability of HCPs to maintain confidentiality, poor quality counselling, healthcare providers’ attitudes, and the inadequacy of clinic processes, were clearly shown to impact their ability to link to care. It is a concern that health system-level barriers to linkage to HIV care were the second most cited factors in this study given the fact that communities rely on the health system structures for HIV care. This finding is in line with other research outcomes [18, 41, 42]. In a recent study in Mozambique by Fuente-Soro et al, the health system was cited to be the most influential barrier to linkage to HIV care since the beginning of the HIV pandemic [41]. Similar findings were recently reported in studies by Sanga et al. [18] and Eshun-Wilson et al, [42].

The quality of counselling received was clearly shown to impact participants’ ability to link to and remain in care. We found that the counselling process in uThukela was centred on the importance of taking medication on time and consistently and missed critical psychological and social effects of discovering one’s HIV-positive status. The counselling included the importance of condom use even with being HIV positive, management of the physical and mental health changes and sources of support, amongst others. Participants reported that in cases where HIV education was provided, it was inconsistent with the guidelines. The counselling quality was also impacted by time, where participants felt the counselling provided left little room for asking questions and discussing concerns, as the counsellor had several patients to attend to. As this was the dominant theme in the study, a whole systems approach to tackling poor counselling is needed. In South Africa, a peer-led case management intervention such as Coach Mpilo has been introduced and has shown high uptake and high retention in HIV care at both the community level and in healthcare facilities among men newly and previously diagnosed with HIV [35]. Coach Mpilo is a variation of the peer mentor or case manager paradigm, in which men living with HIV who have overcome their own obstacles and achieved treatment stability serve as coaches to newly diagnosed men and other men striving to achieve treatment stability [35]. Peer-support models have been shown to play an important role in addressing many barriers associated with treatment interruptions, thereby improving adherence to ART among individuals [43–45]. It could be that the individual factors which were found to be barriers in this study are directly or indirectly linked to poor counselling experience.

Segregation of patients in the facilities was also highlighted as a barrier to linkage to care in this study. Just like in other research findings [46–48], participants in this study did not want to be seen going to collect ARVs. Whilst the role of differentiating patient files and queuing in the facility assists in easing the work of health personnel, alternative routes need to be explored as patients continue to report being ‘othered’ in the facility. This is important in decreasing stigma and improving patient clinic attendance. Although concerns about possible stigma were mentioned by a few participants, it is important to highlight that experienced stigma was not mentioned in this study and this speaks to the country’s massive work in eradicating HIV-related stigma. Participajnts, particularly men, suggested the use of linkage officers, using non-branded cars to deliver medication to their homes instead of them queuing at the health facilities. The Department of Health needs a buy-in from respective care users on how to approach the work of linkage officers bringing treatment to participants' homes, as some patients might choose not to disclose to family members.

### Support from significant others

During pre-and post-test counselling, the importance of having an individual share one’s status is emphasized. Findings from this study showed that disclosure to significant others positively influenced linkage to care. Case managers have been shown to be highly effective in providing counselling services and support to individuals, enabling them to identify inner strengths needed to facilitate linkage and retention to HIV care, resulting in improved health outcomes [49, 50]. This finding highlights the important role played by case managers in supporting patients and families, through counselling, to address disclosure challenges and stigma. Similar findings have been noted in other settings [51]. A systematic review conducted in sub-Saharan Africa also revealed that fear of disclosure was associated with non-linkage to care [17]. Significant others in this study took on multiple roles as counsellors, financial supporters, and adherence officers, filling in a huge gap in the system of care. Hence, HIV education should go beyond focusing on the infected patients, but continued regular community campaigns should be provided, to ensure that families and friends providing support have the updated guidelines on HIV care. It was not surprising that the majority of those who had not disclosed their status did not link to care, as this source of important support was not accessed by these participants.

### Socio-economic factors

Socio-economic factors were also reported as barriers to linking to care. Over 60% of participants in the study were unemployed, therefore access to money to buy food and travel to the clinic were issues. Specifically, food insecurity has been documented to have a huge impact on PLHIV including linkage to care [52, 53]. Food insecurity prevented some participants from linking to care, due to concerns about defaulting. Disclosure could also be of importance to access food in the family, especially for those who mentioned not taking medication due to lack of food. In this study, those who had disclosed their status mentioned how their family needs structure to accommodate them in managing their new health status. Just as the WHO recommended the integration of nutrition interventions into HIV/AIDS treatment and care programmes, the South African government should consider an effective implementation of such interventions.

### Different modes of treatment

Issues with ART extended to the complex relationship between treatment and acceptance of one’s HIV status, and preference for alternative modes of medication. Some of those who linked to care and discontinued, did so due to negative experiences with taking medication, with a few struggling to swallow pills. These are issues that could be dealt with in effective and quality HIV counselling sessions. In addition to the support provided by HIV counsellors, the use of case managers, linkage officers, and nurse clinicians has become critical in providing support to individuals diagnosed with HIV, in so far as complementing the initial counselling services provided by counsellors [33]. Case managers take a comprehensive approach to providing support tailored to the needs of individuals, by recognizing the many aspects that influence a patient’s ability to adhere to HIV treatment [33]. Strengthening health systems in this regard is therefore crucial. Patients from uThukela would greatly benefit from being taught alternative ways of taking pills so that this does not hinder the taking of ARVs. There are clinical trials that are exploring the use of injections instead of pills [54]. Should this be available, some PLHIV such as the ones found in this study, would greatly benefit from this. As a province that is significantly cultural, it is not surprising to find participants who preferred the use of traditional medicine instead of ART [55]. This indicates that conversations with traditional leaders, as far as HIV is concerned, need to continue. Traditional leaders remain influential and command respect in their communities. They are held in high esteem among their followers and can assist in shaping behaviours, including providing support to individuals toward linkage to and retention in HIV treatment. Therefore, their active participation in implementing HIV programmes may contribute to improved HIV uptake, linkage to care, and adherence to ART.

### Impacts of delivering UTT

South Africa has invested so much in the upscaling of UTT in the hope that many PLHIVs will link and remain in care. This study is one of the very few that was conducted amongst PLHIV to understand their views and perceptions about the impact of delivering UTT. Participants in this study seemed to generally accept the implementation of UTT. Few seem to prefer the old guidelines of waiting for their CD4 count to decrease before starting treatment. The acceptance of UTT came with the understanding that starting treatment immediately would give HIV-positive clients a better chance to live longer. The positive views on the implementation of UTT were similarly found in other studies [56–58].

#### Study limitations

The use of Life-story as a data collection approach could have been beneficial in unearthing deep reflections during the interview process. Most interviews were conducted in clinics, which might have caused discomfort and led to socially desirable responses. However, interviewers were well-trained in managing these issues. Also, we did not include peadiatric patients and caregivers in the sample.

This cohort study has presented an overview of the demographics and context of the participants, and it demonstrates that the linkage to HIV care rate is high among the HIV-positive cohort in the uThukela District. This could have been influenced by our field team, who provided educational materials about linkages and support to the participants. This is important to inform the investigations and the analysis of barriers and enablers of linkage to and retention in care experience of the HIV-positive cohort. Lastly, the results may not be generalizable as this study included a small sample size. Data presented are from a district in KZN province, which may be different from the other eight provinces in South Africa.

## Conclusion and next steps

For a country such as South Africa, health system-level interventions like UTT would need to focus on clinic readiness in terms of providing patients with the necessary and effective health services such as proper counselling care and psychological assistance to manage their HIV status. This would involve an investment into both quantity and quality of lay counsellors in health care facilities, which would improve the number of patients who link to care. This would ensure that the linkage process is completed even by those who had negative experiences during HIV testing services. This study highlighted issues with UTT acceptance by PLHIV. More studies are needed on the acceptability of UTT by healthcare providers in South Africa. Voluntary counselling and testing (VCT) and the benefits of universal test and treat (UTT) need to be emphasized in the district. This will ensure that not only testing targets are met but also viral suppression targets.

This study showed that most participants were youth, therefore initiation of youth clinics and youth mobile clinics is an important step in the district, in order to ensure that this group is retained in care. The role of a digital application in responding to patient concerns would be beneficial in this setting, as most live far from the clinic, with limited access to transport. Community-based ART initiation services and mobile clinic services are effective in improving access to HIV treatment in sub-Saharan Africa, as it address numerous distinctive barriers to accessing HIV services in clinic settings. Therefore, scaling of these strategies is critical. Finally, interventions to improve linkage to and retention in HIV care could consider a holistic approach, which should not only focus on the provision of medication for PLHIV, but also on the provision of psychological, social, and financial support services, as the next step for intervention planners.

## Data Availability

The datasets generated and/or analysed during the current study are available from the corresponding author on reasonable request.
